# Association between autoimmune thyroiditis and clinicopathological features of papillary thyroid carcinoma: a prospective study

**DOI:** 10.20945/2359-4292-2026-0090

**Published:** 2026-08-05

**Authors:** Amira Bouchenna, Abdelghani Tibouk, Brahim Ghennam, Khawla Boumaraf, Samia Ould Kablia, Meriem Bensalah

**Affiliations:** 1 Endocrinology unit, Central Hospital of Army, Algiers, Algeria; 2 Pathology unit, Central Hospital of Army, Algiers, Algeria; 3 Nuclear medicine unit, Central Hospital of Army, Algiers, Algeria; 4 Epidemiology unit Beni Messous Hospital, Algiers, Algeria

**Keywords:** Papillary thyroid carcinoma, autoimmune thyroid disease, Hashimoto’s thyroiditis, anti-thyroglobulin antibodies

## Abstract

**Objective:**

This study aimed to evaluate the impact of coexisting autoimmune thyroiditis (HT) on the clinicopathological characteristics and prognosis of patients with papillary thyroid carcinoma (PTC).

**Subjects and methods:**

This prospective study included patients diagnosed with papillary thyroid carcinoma who underwent thyroid surgery. Patients were divided into two groups according to the presence or absence of histologically confirmed thyroiditis. Demographic data, tumor characteristics, pathological features, and postoperative outcomes were analyzed. Statistical comparisons were performed to assess differences between the two groups.

**Results:**

Thyroiditis was observed in a significant proportion of patients with PTC. Patients with associated thyroiditis showed smaller tumors and more favorable clinicopathological characteristics compared to those without thyroiditis. However, no significant differences were observed in major oncological outcomes. Recurrence rates were lower in the thyroiditis group. The presence of thyroiditis was associated with more favorable prognostic features.

**Conclusion:**

The coexistence of thyroiditis in patients with PTC was associated with more favorable clinicopathological features. However, no significant differences were observed in major oncological outcomes, and the short follow-up period limits the assessment of long-term prognosis. These findings suggest an association with less aggressive initial presentation rather than a definitive prognostic impact.

## INTRODUCTION

Thyroid cancers account for approximately 1% of all malignancies and represent the most common endocrine cancers, of which 85-90% correspond to papillary thyroid carcinomas (PTCs). Their incidence has been steadily increasing in recent years, largely due to improved, more widely accessible diagnostic modalities, including cervical ultrasound and fine-needle aspiration cytology. Despite this rise in incidence, mortality remains low, with a 5-year survival rate exceeding 90% (^1^). In Algeria, thyroid cancer ranks as the third most common cancer among women (^2^).

Autoimmune thyroid disease is the most frequent autoimmune disorder and is represented by Hashimoto’s thyroiditis and Graves’ disease, with an estimated incidence of approximately 5% in the general population (^3^).

The association between PTC and autoimmune thyroiditis (HT) was first described in 1955 by Dailey (^4^). However, the relationship between these two conditions remains complex and not fully understood. Several mechanisms have been proposed, including chronic inflammation, immune-mediated effects, and the role of thyroid-stimulating hormone, although their exact contributions to tumor development and progression remain debated (^5^).

The aim of this study was to investigate the associations between HT and clinicopathological features, as well as early treatment outcomes, in patients with PTC.

## SUBJECTS AND METHODS

We conducted a prospective longitudinal study including 267 patients over the age of 18 years who were evaluated at a specialized referral center and diagnosed with PTC between January 2020 and December 2022.

The diagnosis of autoimmune thyroiditis was established on the basis of histopathological examination. After determining the prevalence of thyroiditis in the cohort patients were divided into two groups: those with thyroiditis and those without. The primary goal of the study was to assess the association between the presence of autoimmune thyroiditis and tumor size, which is a key clinicopathological feature.

Secondary outcomes included the evaluation of the association between thyroiditis and other prognostic factors, such as lymph node metastases, extrathyroidal extension, vascular invasion, multifocality, and risk stratification according to the TNM/AJCC 2017 (^6^) and ATA 2015 (^7^) classifications. Early treatment outcomes, including response to therapy and recurrence at 9 months, were also analyzed.

Given the increase in early diagnosis of PTC and the consequent reduction in tumor size, in addition to the classical prognostic threshold of 4 cm, a secondary stratification at 2 cm was applied to compare the two groups.

**Figure 1:**
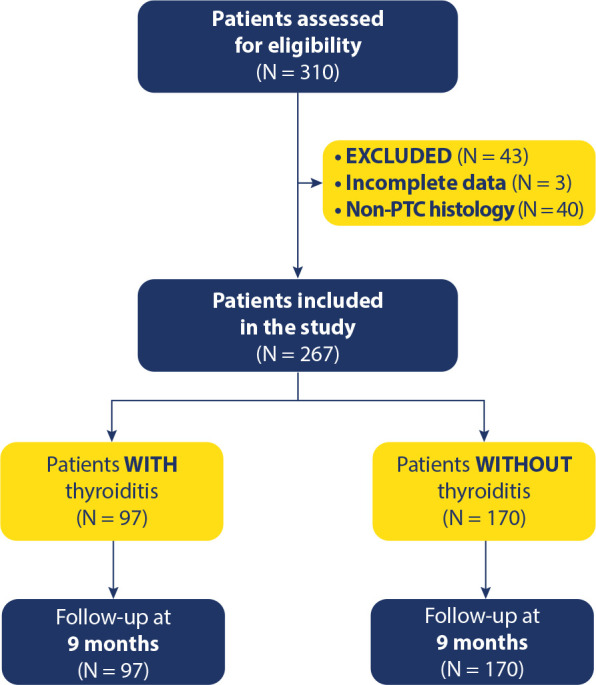
Flow diagram of patient selection and study design.

The exclusion criteria included patients with incomplete prior clinical data who had previously undergone thyroid surgery, or those who did not have papillary thyroid carcinoma. Potential sources of bias, including selection bias and the relatively short follow-up period, were considered when interpreting the results. A flow diagram of patient inclusion was constructed in accordance with the STROBE recommendations (**Figure 1**).

Statistical analyses were performed using SPSS version 27. Continuous variables were expressed as the means ± standard deviations, and categorical variables were expressed as frequencies and percentages. The normality of continuous variables was assessed prior to analysis. Depending on the data distribution, parametric or non-parametric tests were applied.

Comparisons between qualitative variables were performed using the chi-square test or Fisher’s exact test when appropriate. Continuous variables were compared using Student’s t test or non-parametric equivalents.

Variables with a p value < 0.2 in univariate analysis were included in a multivariate logistic regression model. The dependent variable in the multivariate analysis was the presence of thyroiditis. Odds ratios (ORs) with 95% confidence intervals (CIs) were calculated. Statistical significance was set at p < 0.05.

No adjustment for multiple comparisons was performed; therefore, and the results should be interpreted with caution.

The study was conducted in accordance with internationally accepted ethical principles, and written informed consent was obtained from all participants prior to inclusion.

## RESULTS

A total of 267 patients with papillary thyroid carcinoma (PTC) were included. There was a marked female predominance, with 201 women (75.3%) and 66 men (24.7%). The mean age at diagnosis was 44.8 ± 0.8 years (range: 20-84 years). A family history of thyroid cancer was reported in 7 patients (2.6%).

The mean initial serum TSH concentration was 2.44 ± 0.15 IU/L (range: 0-27.49 IU/L). TSH values were within normal limits in 81.2%, low in 8.6%, and elevated in 10.2% of patients.

All patients underwent total thyroidectomy. Lymph node dissection was performed in 52.8% of cases, among whom 74.5% underwent prophylactic central dissections and 25.5% underwent therapeutic dissections including lateral compartments.

According to the 2017 TNM classification, 67% of tumors were staged T1 (including 31.8% microcarcinomas), 22.5% T2, and 10.2% T3-T4. One case of non-invasive follicular thyroid neoplasm with papillary-like nuclear features (NIFTPs) was identified (3.5 cm). Among patients who underwent lymph node dissection, 12% had lateral lymph node metastases, 7.1% had central metastases, and 33.7% had no metastatic involvement. Preoperatively, one patient presented with bone metastasis. Post-therapeutic scintigraphy revealed distant metastases in 5 patients (pulmonary, mediastinal, or clavicular), accounting for 2.24% of the cohort.

Based on the 2015 ATA recurrence risk stratification, 76% of the patients were categorized as low risk, 12.4% as intermediate risk, and 11.6% as high risk.

Radioiodine therapy was administered in 80.1% of cases. It was not indicated in 19.9%, predominantly for unior multifocal micro-PTCs with a cumulative tumor burden ≤ 1 cm and the absence of capsular invasion or vascular emboli.

At the nine-month follow-up, the treatment response, according to the ATA 2015 criteria, was as follows: 69.9% had an excellent response, 15.1% had an incomplete biochemical response, 7.5% had an incomplete structural response, and 7.5% had an indeterminate response.

Recurrence was documented in 22 patients (8.24%): 68.2% presented with cervical lymph node metastases, 9.1% developed distant metastases, and 22.7% demonstrated disease progression without identifiable lesions on ultrasound, CT, or FDG-PET. Management included surgery followed by radioiodine in 7 patients and a second radioiodine course in 15 patients. One patient was deemed inoperable due to local invasion and was not eligible for tyrosine kinase inhibitors. Another patient was classified as radioiodine refractory after a second treatment and initiated sorafenib treatment.

Histopathological features of chronic autoimmune thyroiditis were present in 97 patients (36.3%).

When comparing prognostic indicators between patients with and without thyroiditis, several statistically significant associations were identified. PTC occurring in the setting of thyroiditis was more common in women (p < 0.001) and in younger patients (p = 0.015). The mean tumor size was significantly smaller in the thyroiditis group (1.58 ± 1.1 cm vs. 1.78 ± 1.27 cm; p < 0.001), and multifocality was more common (p = 0.043).

Conversely, no significant differences were found for capsular invasion (p = 0.79), extrathyroidal extension, vascular emboli (p = 0.20), aggressive histologic variants (p = 0.36), lymph node metastases (p = 0.49), or distant metastases (p = 0.67). Likewise, the ATA 2015 (p = 0.41), TNM 2017 (p = 0.38), and AJCC staging (p = 0.74) did not significantly differ between the two groups.

Several ultrasound and biochemical parameters differed significantly. TSH levels were higher in patients with thyroiditis (3.21 ± 0.37 IU/L vs. 2.01 ± 0.48 IU/L; p < 0.001). The mean nodule size on ultrasound was smaller (1.82 ± 0.1 cm vs. 2.4 ± 0.1 cm; p = 0.001), and the EU-TIRADS 5 classification was more prevalent (71.1%; p = 0.03) in patients with thyroiditis. Thyroid volume also differed significantly (p = 0.017) (^8^). No difference was observed according to the 2017 Bethesda system (p = 0.92) (^9^).

Radioiodine indications (p = 0.68) and administered activities (p = 0.10) did not differ between the two groups. The mean thyroglobulin levels at ablation were significantly lower in the thyroiditis group (12.54 ± 8.04 ng/mL vs. 61.65 ± 38.04 ng/mL; p < 0.001), while anti-Tg antibody positivity showed no statistical difference (p = 0.70).

At the nine-month assessment, the response to therapy was significantly associated with the presence of thyroiditis (p < 0.001). Notably, 18% of patients with thyroiditis were classified as having an indeterminate response. However, the nine-month recurrence rates did not differ significantly between the groups (p = 0.92).

All outcomes are summarized in **Table 1**.

**Table 1:** Clinicopathological characteristics according to the presence of autoimmune thyroiditis (HT)

Variable	HT (n = 97)	No HT (n = 170)	p value
Female sex, n (%)	85 (87.6)	116 (68.2)	< 0.001
Age, years (mean ± SD)	42.8 ± 1.1	46.2 ± 1.0	0.015
Tumor size, cm (mean ± SD)	1.58 ± 1.1	1.78 ± 1.27	< 0.001
Tumor size > 2 cm, n (%)	17 (^18^)	50 (^29^)	0.03
Multifocality, n (%)	34 (35)	40 (^24^)	0.043
Capsular invasion, n (%)	22 (22.7)	41 (24.1)	0.79
Vascular emboli, n (%)	21 (21.6)	49 (28.8)	0.20
Lymph node metastasis (yes), n (%)	18 (^33^)	33 (38)	0.49
TNM stage (advanced stage vs. low stage), n (%)	9 (9.2) 88 (90.8)	18 (10.7) 151 (89.3)	0.38
Aggressive histologic variants, n (%)	5 (^5^)	5 (^3^)	0.36
ATA high-risk category, n (%)	9 (9.3)	22 (12.9)	0.41
Radioiodine therapy (yes), n (%)	79 (81.4)	135 (79.4)	0.68
Recurrence at 9 months, n (%)	8 (^9^)	14 (9.3)	0.92

ATA: American Thyroid Association.

Low-stage tumors corresponded to T1-T2 tumors, and advanced-stage tumors corresponded to T3-T4 tumors according to the 2017 TNM classification. Lymph node metastasis analysis was restricted to patients who underwent lymph node dissection.

Multivariate binary logistic regression including, variables with p values < 0.2 in univariate analysis, revealed the following independent prognostic factors: age > 55 years, female sex, tumor size > 2 cm, and the presence of vascular emboli. The results are summarized in **Table 2**.

**Table 2:** Multivariate logistic regression analysis of factors associated with the presence of Hashimoto’s thyroiditis

Variable	Adjusted OR	95% CI	p value
Female sex	3.59	1.43-8.98	0.006
Age >55 years	0.35	0.13-0.97	0.043
Tumor size >2 cm	0.38	0.15-0.98	0.046
Vascular emboli	0.39	0.16-0.94	0.036
Multifocality	-	-	0.11
Lymph node metastasis	-	-	0.60
Capsular invasion	-	-	0.60

“-” : not retained in the multivariate model.

## DISCUSSION

The association between papillary thyroid carcinoma (PTC) and autoimmune thyroiditis (HT) has been documented widely since its first description in 1955 (^4^). However, interpretation remains challenging due to substantial methodological heterogeneity among published studies, most of which are retrospective (^10^-^12^).

The relationship between HT and PTC remains complex and not fully understood. Elevated thyroid-stimulating hormone (TSH) levels, chronic inflammation, and cytokine-mediated pathways have been suggested to contribute to tumor development. However, their clinical implications remain debated, with recent studies highlighting the potential dual role of autoimmune processes in both tumor initiation and the modulation of tumor behavior (^13^,^14^).

In addition, the frequency of BRAFV600E mutations appears to be lower in PTC cases associated with HT. Even when present, autoimmune mechanisms may partially counterbalance their oncogenic effect, which could contribute to the more favorable clinicopathological features often observed in this subgroup (^13^).

In line with previous series, our cohort demonstrated a marked female predominance, likely reflecting hormonal influences on immune modulation and tumorigenesis. Moreover, patients with HT were younger at diagnosis, which is a well-established favorable prognostic factor in contemporary staging systems (^15^).

Histopathologically, tumors were significantly smaller in the HT group, which is consistent with most published data (^11^,^12^). Multifocality was also more frequent, which may reflect a field-effect phenomenon rather than increased tumor aggressiveness (^13^,^16^).

An important explanation for the association between HT and smaller tumor size may be detection bias, as patients with HT are more likely to undergo clinical and ultrasound monitoring, leading to earlier diagnosis. However, this effect should be interpreted with caution, as a substantial proportion of patients in our cohort were euthyroid and did not undergo systematic surveillance.

We did not observe significant differences in vascular invasion, lymph node metastases, extrathyroidal extension, ATA risk stratification, or TNM staging, in agreement with several previous studies reporting inconsistent or non-significant associations (^17^-^20^). Recent large-scale analyses have also highlighted heterogeneous results regarding the relationship between thyroid autoantibodies and tumor aggressiveness (^21^).

Radioiodine use and administered activities were comparable between groups, consistent with findings from the previous studies (^22^-^25^).

Thyroglobulin levels at the time of ablation were significantly lower in patients with HT; however, these findings should be interpreted with caution due to the known interference of anti-thyroglobulin antibodies, which may lead to the underestimation of Tg levels (^26^). Recent prospective data further support the clinical relevance of thyroid autoantibodies in both diagnostic assessment and risk stratification in thyroid cancer (^27^).

At the 9-month follow-up, patients with HT were more likely to demonstrate an excellent response but were also more likely to be classified as having an indeterminate response. This pattern likely reflects the impact of autoimmune status on thyroglobulin and antibody kinetics rather than true differences in disease status. This ambiguity raises concerns regarding the applicability of the ATA indeterminate response category in this subgroup. Similar observations have been reported in the literature, with a high proportion of indeterminate responses subsequently reclassified as excellent responses during longer follow-up (^28^-^30^).

De Leo and cols. reported a higher frequency of incomplete biochemical response, likely related to residual thyroid tissue or abnormalities in thyroglobulin measurements, without necessarily affecting the structural aggressiveness of the cancer (^31^).

No significant association was observed between HT and early recurrence, consistent with findings from several previous studies (^24^,^32^,^33^). However, the short follow-up period limits the interpretation of long-term outcomes.

From a clinical perspective, the assessment of treatment response in patients with HT remains challenging because of the interference of anti-thyroglobulin antibodies. The higher rate of indeterminate responses observed in this subgroup may therefore reflect biochemical limitations rather than persistent disease. These findings suggest that early reassessment at 9 months, particularly when based on thyroglobulin levels, may lead to potential misclassification. In selected patients with positive anti-thyroglobulin antibodies, delaying assessment of response until anti-thyroglobulin antibodies become negative may help avoid misclassification and unnecessary therapeutic escalation, although this hypothesis requires confirmation in larger prospective studies.

This study has several limitations. Its single-center design may limit generalizability, although its prospective nature represents a strength, allowing for standardized data collection. The relatively short follow-up (9 months) does not allow for reliable assessment of long-term outcomes in this typically indolent disease. In addition, the association between HT and smaller tumor size may be partially influenced by detection bias, although many patients in our cohort were euthyroid and not routinely monitored. Finally, the interpretation of thyroglobulin levels remains limited by antibody interference, and residual confounding cannot be excluded given the study’s observational design.

In conclusion, in this prospective study, autoimmune thyroiditis was associated with certain favorable clinicopathological features, including younger age at diagnosis and smaller tumor size. However, no significant differences were observed in key oncological outcomes, such as lymph node metastasis, extrathyroidal extension, staging, or recurrence, at 9 months.

These findings suggest that autoimmune thyroiditis may be associated with a less aggressive initial presentation of papillary thyroid carcinoma rather than a definitive prognostic impact.

Given the relatively short follow-up period and the potential influence of detection bias, these results should be interpreted with caution. Further prospective studies with longer follow-up periods are needed to better clarify the relationship between autoimmune thyroiditis and long-term outcomes in papillary thyroid carcinoma.

## Data Availability

the authors are willing to share all data related to the study upon reasonable request.
